# From Structural Design to Molecular Mechanisms: The Evolution of Solar Evaporators

**DOI:** 10.1021/cbe.5c00110

**Published:** 2025-12-09

**Authors:** Dong Wu, Jie Zhu, Qichen Zhang, Xiayun Huang, Zhihong Nie

**Affiliations:** The State Key Laboratory of Molecular Engineering of Polymers and Department of Macromolecular Science, 12478 Fudan University, Shanghai 200438, People’s Republic of China

**Keywords:** Solar Evaporation, Polyelectrolyte, Osmotic Pressure Difference, Noncovalent Interactions, Thermal Management

## Abstract

To address global water scarcity, solar-driven interfacial evaporation has emerged as a promising solution that minimizes energy consumption and environmental impact by harvesting solar energy directly at the air–water interface. Recent advances show that performance breakthroughs depend on the synergistic interplay between macroscopic structural designs and molecular-level mechanisms. This review traces the evolution of solar evaporators, from buoyancy-driven floating architectures and enhanced water transport enabled by capillary and osmotic pressure differences, to state-of-the-art regulation of water association states through hydrophobic interaction, hydrogen-bonding, and electrostatic interaction. These strategies accelerate mass transport, optimize solar energy utilization, lower evaporation enthalpy, and enhance long-term stability, achieving water transport rates up to 0.091 g min^–1^, evaporation rates up to 6.92 kg m^–2^ h^–1^ under 1 kW m^–2^ illumination, and salt rejection efficiencies above 99.99%. Despite these advances, challenges remain in achieving a precise mechanistic understanding of interfacial evaporation, scaling up fabrication, and standardized performance evaluation. This review highlights these issues and outlines future research directions to accelerate the practical deployment of solar-driven interfacial evaporation technologies for sustainable desalination and water purification.

## Graphical abstract

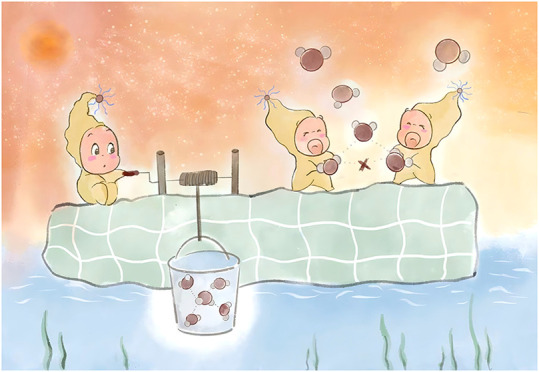

## 1 Introduction

By 2050, over 840 million people are projected to face chronic water scarcity.^1,2^ Conventional water treatment technologies, such as membrane-based separation and thermal distillation, remain highly energy-intensive, reliant on fossil fuels, and pose risks of secondary pollution.^3–5^ In contrast, solar-driven interfacial evaporation has emerged as a sustainable alternative by directly harvesting solar energy at the air–water interface. High-performance solar-driven interfacial evaporation systems maximize light absorption and photothermal conversion, minimize heat loss to the bulk water, and lower evaporation enthalpy by modulating water association states.^6,7^ After nearly a decade of research, solar-driven interfacial evaporation systems approached water transport rates up to 0.091 g min^–1^,^8^ achieving evaporation rates up to 6.92 kg m^–2^ h^–1^ under 1 kW m^–2^ illumination,^9^ and salt rejection efficiencies exceeding 99.99%.^8^

To realize efficient solar-driven interfacial evaporation, three aspects of structural and molecular design are particularly critical. First, the floating evaporator architecture provides the foundation of operational reliability by ensuring effective light capture and stable air–water interface contact.^10,11^ Second, water transport often constitutes the rate-limiting step in evaporation. Synergistic strategies, such as graded capillary channels^12–14^ for pressure-driven flow and polyelectrolyte gradient^8^ for osmotic pumping, are essential to overcome gravitational and viscous resistance. Third, evaporative surface functionalization governs both energy harvesting and consumption by regulating interfacial light absorption, photothermal conversion, thermal confinement, and evaporation enthalpy.^15–17^ In particular, noncovalent interactions—including hydrophobic interaction, hydrogen-bonding, and electrostatic interaction—reshape water association states to lower the cohesive energy of vaporization, with polyelectrolytes playing a central role in reducing evaporation inter and enhancing performance.

In this review, we summarize the integrated pathways through which structural design and molecular interactions drive the exceptional performance of solar-driven interfacial evaporator. We evaluate their effectiveness within the framework of floating capability, water transport, energy harvesting, and interfacial regulation, highlighting both achievements and limitations. Finally, we discuss the remaining challenges and propose future directions to accelerate the translation of solar-driven interfacial evaporator from laboratory demonstration to real-world application.

## 2 Floating Evaporator Designs

In solar-driven interfacial evaporation, evaporator buoyancy is a fundamental requirement for operational reliability. Typically, two design solutions are used to obtain floating capability (Figure 1): (1) fabricating monolithic evaporators with densities similar to that of water, allowing for self-floating capability; and (2) utilizing auxiliary floating platforms to support the evaporator. Furthermore, buoyancy must work together with thermal insulation and efficient water transport to maintain high evaporation performance.

**Figure 1:**
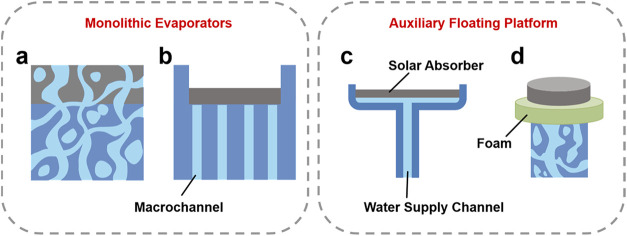
Schematic illustration of monolithic evaporators and auxiliary floating platform. (a) A monolithic bilayer evaporator with open- and closed-cell porous structures enabling self-buoyancy. (b) The wick-free monolithic evaporator with vertically aligned water channels and photothermal black-paint coating on the top surface. (c) Water-lily inspired evaporator supported by a polystyrene foam floater with a copper foam photothermal layer on top. (d) Life-buoy-inspired evaporator, in which a floating foam ring encircles a central water supply and photothermal layer.

### 2.1 Monolithic Evaporators

Gang Chen’s group^18^ developed a monolithic bilayer evaporator in 2014, consisting of a graphite photothermal layer on the top of a porous carbon foam floating layer, reaching an impressive 85% solar-to-vapor conversion efficiency under 10 kW m^–2^ illumination (Figure 1a). The closed pores in the carbon foam captured air, lowering the overall density, allowing density matching with water, and ensuring stable floating. Meanwhile, the interconnecting open pores functioned as water channels, allowing continuous water transport to the evaporation surface. Inspired by this pioneering concept, a number of monolithic evaporators made of low-density porous materials have been proposed.^11,19–21^ Among them, Evelyn Wang’s group^22^ developed a wick-free monolithic evaporator using a polyurethane foam substrate with vertical macrochannels (2.5 mm in diameter) drilled through the foam and a photothermal black-paint coating on the upper surface (Figure 1b). Convective flow inside the channels effectively permitted salt rejection with minimal additional heat losses, and the vertically aligned channels produced a contained water layer that allowed for continuous water delivery. The evaporator was able to operate steadily for a week in brine (20 wt % salinity), with an average evaporation rate of 1.1 kg m^–2^ h^–1^. Nevertheless, the production of such monolithic buoyant materials remains challenging, and their evaporation efficiency is often limited.

### 2.2 Evaporators with Auxiliary Floating Platforms

To address these limitations, auxiliary floating platforms have been widely adopted.^23,24^ Jia Zhu’s group^25^ designed a two-dimensional evaporator based on the structure of water lily leaves, with a copper foam photothermal layer supported by a low-density polystyrene (PS) foam floater (Figure 1c). After 18 days of sustained operation in brine (10 wt % salinity), the device obtained an average evaporation rate of 1.27 kg m^–2^ h^–1^ and ∼80% efficiency under 1 kW m^–2^ illumination. Liping Heng’s group^26^ employed PS foam support modeled after life buoys, in which a floating foam ring encircles a central water supply and photothermal layer (Figure 1d). Based on this configuration, they created a corn-cob/carbon nanotube (CNT) composite evaporator and a cellulose/CNT composite evaporator, both of which exhibited steady operation with evaporation rates of 3.06 and 2.56 kg m^–2^ h^–1^, respectively. In fact, both monolithic evaporators and auxiliary floating evaporators exhibit comparable floating stability and maintain reliable buoyancy. In the auxiliary floating design, low-density foam is inserted between the evaporative surface and the bulk water. The trapped stationary air within the foam provides effective thermal insulation and significantly reduces heat transfer to the underlying water. Consequently, this configuration delivers superior insulation performance compared with monolithic structures. Nevertheless, monolithic evaporators, owing to their simpler integrated architecture, are more amenable to large-scale fabrication and practical deployment.

## 3 Water Transportation

According to mass conservation, the water transport rate in an interfacial evaporator must be equal to or greater than the evaporation rate in order to maintain steady vapor production. With the advancement of evaporation surface engineering,^27,28^ intrinsic water transport capacity has emerged as a critical bottleneck in sustaining the required evaporation flux. Recent studies have introduced hierarchical structures, which offer promise for optimizing continuous water delivery.^4,8,29^ Within such structures, two primary mechanisms come into play: capillary-driven transport and osmotic pressure-driven transport.^18,30^

### 3.1 Capillary-Driven Transportation

Capillarity refers to the spontaneous upward movement of liquid in narrow and wettable channels, driven by surface tension and intermolecular interactions.^31^ This mechanism, widely observed in plants, provides an additional driving force for water transport. The capillary pressure (Δ*P*_cap_) can be estimated using the Young–Laplace equation^32^

1 $${\Delta P}_{\mathrm{cap}}=\frac{2\gamma \cos\left(\theta -\alpha \right)}{r}$$

where γ is the surface tension of water (72 mN m^–1^), θ is the water contact angle (°) with the channel wall, α is the angle (°) between the pore wall and the vertical direction, which is positive for a top-narrow, bottom wide channel, and *r* is the radius (m) of the pore channel at the meniscus. For a superhydrophilic vertical channel (θ ≈ 0° and α = 0°), cos­(θ-α) reaches its maximum value of 1. Under this condition, capillary pressure depends solely on the pore size, being inversely proportional to *r*; in other words, smaller pores generate stronger capillary forces. However, water transport is simultaneously limited by viscous resistance, which can be quantified by the Hagen–Poiseuille equation^33^

2 $${\Delta P}_{\mathrm{visc}}=\frac{8\mu h}{\pi r^{4}}Q$$

where μ is the dynamic viscosity of water (1 mPa s), *h* is the channel length (m), and *Q* is the volumetric flow rate (m^3^ s^–1^). Notably, viscous resistance scales inversely with the fourth power of the channel radius, implying that excessively small pores, while generating strong capillary forces, also impose substantial hydraulic resistance. Therefore, rational design requires balancing capillary driving pressure with viscous losses to optimize water transport efficiency.

Beyond pore size, pore geometry plays a decisive role in governing water transport. Vertically aligned channels (Figure 2a), owing to their ordered arrangement and straight orientation, significantly reduce tortuosity and viscous resistance, enabling rapid, straight-line water delivery. In contrast, tapered gradient channels, where the pore diameter gradually varies along the transport direction (i.e., top-narrow, bottom wide channel, Figure 2b), establish a continuous capillary pressure gradient for directional water delivery according to the Young–Laplace relation,^13,35^ in which the angle α between the pore wall and the vertical direction influences the effective pressure. This geometry promotes unidirectional, diode-like water transport with high efficiency. For example, Xi Shen’s group^13^ reported that tapered gradient-channel evaporators achieve a water transport rate of ∼0.072 g min^–1^, compared with 0.049 g min^–1^ in vertical channels—an enhancement of ∼17%, highlighting the potential yet limited improvement of tapered channels. However, most practical channels in the evaporator are composed of random porous networks (Figure 2c). These networks, characteristic of materials such as gels, foams, and sponges, feature highly irregular and branched pore structures. While such structures offer high surface area and porosity, their irregular connectivity results in long, tortuous pathways and local void formation, which collectively impede water delivery.^36,37^ Nevertheless, random porous materials are still widely adopted owing to their facile fabrication, low cost, and broad applicability.

**Figure 2:**
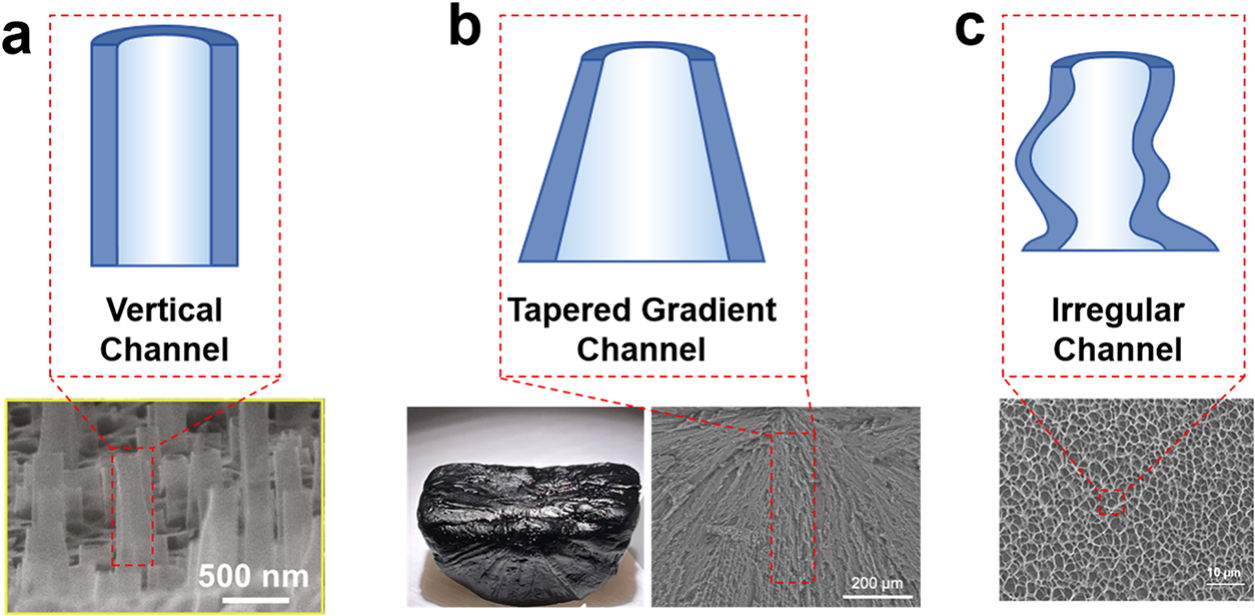
(a) Schematic illustration of a vertical channel and corresponding SEM image of vertically aligned carbon nanotubes (CNTs) channels. Reproduced with permission from ref 34. Copyright 2024, American Association for the Advancement of Science. (b) Schematic illustration of a tapered gradient channel and corresponding optical image and SEM image of a structurally graded aerogel. Reproduced with permission from ref 13. Copyright 2024, Springer Nature. (c) Schematic illustration of an irregular channel and corresponding SEM image of a porous gel. Reproduced with permission from ref 7. Copyright 2019, American Association for the Advancement of Science.

### 3.2 Osmotic Pressure-Driven Transportation

In addition to capillary-driven water transport, osmotic pressure is an important driving force that must be carefully considered. Osmotic pressure arises from an imbalance in chemical potential, acting as a driving force that directs water from regions of lower solute concentration to regions of higher concentration.^38^ Inspired by passive transport in biological cells, researchers have introduced polyelectrolytes into evaporators to generate osmotic gradients.^24,39,40^ The osmotic pressure difference (ΔΠ) between a polyelectrolyte solution and pure water is often estimated using the van’t Hoff equation^41^

3 ΔΠ=iCRT

where *i* is the van’t Hoff factor, which is defined as the ratio of the number of solute species in solution to the number of solute units; it has no dimension and represents the number of dissociated species per solute unit. *C* is the solute’s molar concentration (mol L^–1^), *R* is the ideal gas constant (8.314 J mol^–1^ K^–1^), and *T* is the absolute temperature (K). This expression applies to ideal dilute solutions, where osmotic pressure increases linearly with solute concentration. However, in complex systems, such as heterogeneous polyelectrolyte hydrogels, osmotic pressure can be influenced by solute type, ionic strength, and local concentration variations, leading to significant deviations from the van’t Hoff prediction.

In heterogeneous polyelectrolyte hydrogels, the charge density is substantially higher than in dilute solutions, and the surrounding mobile ions provide strong electrostatic shielding for the charged polymer chains. Consequently, the effective van’t Hoff factor may decline to as low as ∼10% of the theoretical value. Using the uncorrected van’t Hoff equation generally leads to a pronounced overestimation of the osmotic pressure differential. Therefore, for polyelectrolyte systems, osmotic pressure is more accurately predicted from the local ionic concentrations^8^

4 $$\Pi \left(y\right)=k_{B}\left(n_{+}+n_{-}\right)T-\Pi_{0}$$

where Π_0_ = 2*n*_s_ *k*_B_ *T* is the osmotic pressure (Pa) of the bulk solution,^42^ *k*_B_ is the Boltzmann constant (J K^–1^), (*n*_+_ + *n*_–_) represents the sum of the local cation and anion concentrations (m^–3^), and *T* is the absolute temperature (K). These ionic concentrations can be determined by solving the Poisson–Boltzmann equation

5 $$\frac{d^{2}\psi }{dy^{2}}=\frac{e\left[-n_{+}+n_{-}+\varphi n_{p}\left(y\right)\right]}{\varepsilon_{0}\varepsilon_{r}}$$

with boundary conditions *d*ψ/*dy*|_*y*=0_ = 0 and *d*ψ/*dy*|_*y*=–*D*_ = 0, where ψ denotes the electrostatic potential (V), *e* is the elementary charge (C), ε_0_ and ε_*r*_ are the vacuum permittivity (F m^–1^) and relative dielectric constant of the medium, respectively, φ is the charge fraction of polyelectrolyte chains (typically ∼ 10%), and *n*_*p*_(*y*) describes the spatial distribution of polymer chains­(m^–3^), and *D* represents the penetration depth (m) of the polyelectrolyte chains in the hydrogel.

Building on this principle, introducing polyelectrolytes into hydrogels establishes a high osmotic pressure difference that can drive water transport. For example, Renkun Chen’s group^18^ developed a sodium poly­(acrylic acid) (PAANa)-based photothermal hydrogel evaporator with 20 wt % PAANa, which achieved continuous and stable solar desalination with an evaporation rate of 1.3 kg m^–2^ h^–1^ under 1 kW m^–2^ illumination. However, bulk hydrogels, despite providing an osmotic driving force, possess a uniform chemical potential that primarily promotes water migration from the bulk solution into the hydrogel matrix, while limiting efficient transport to the evaporation interface. To overcome this limitation, our group^8^ first proposed the concept of gradient osmotic pressure to enhance water transport not only from the bulk solution into the hydrogel but also from the hydrogel to the evaporation interface. Compared with bulk hydrogels (Figure 3), gradient hydrogels exhibited a 185% increase in water transport rate, generating an osmotic pressure difference of up to 125 kPa and a water flux of 0.091 g min^–1^. In contrast, bulk hydrogels with the same polyelectrolyte content but a uniform osmotic potential achieved only 0.032 g min^–1^. As a result, the gradient hydrogel exhibited a high evaporation rate of 4.5 kg m^–2^ h^–1^ under 1 kW m^–2^ illumination, demonstrating the effectiveness of gradient osmotic pressure in enhancing water transport.

**Figure 3:**
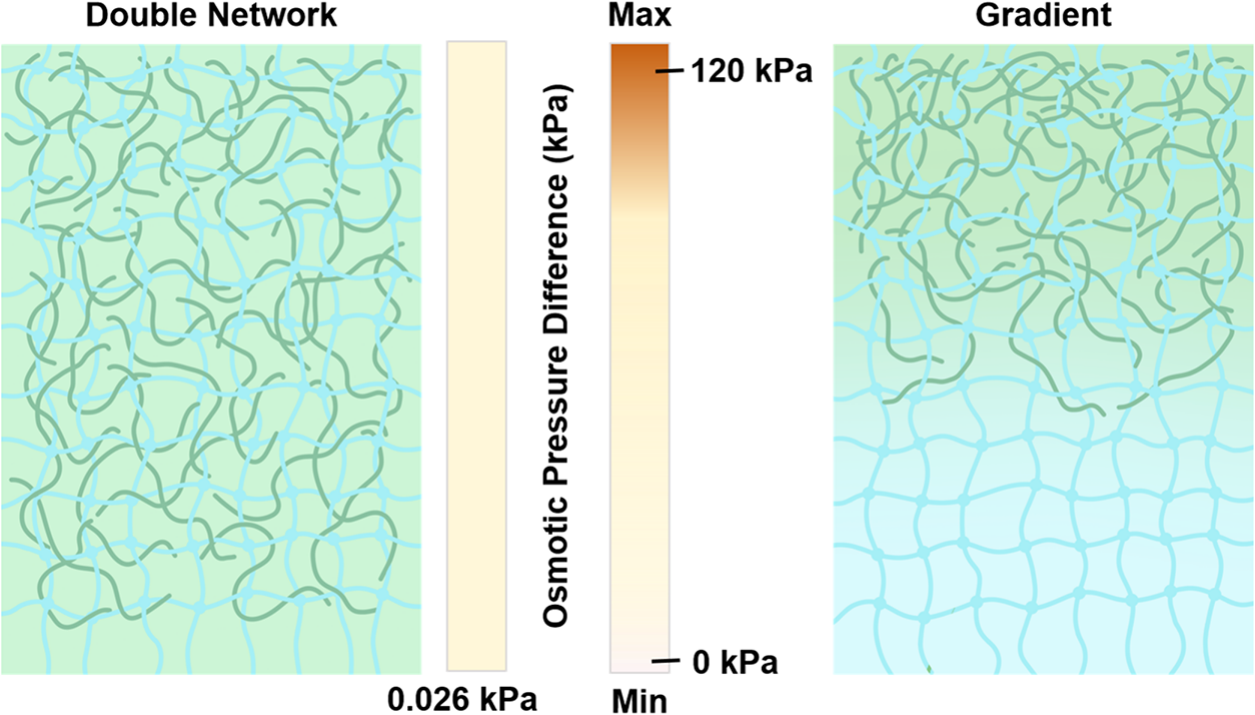
Comparison of osmotic pressure distribution between bulk hydrogel with uniform polyelectrolyte content and gradient hydrogels with a polyelectrolyte concentration gradient.^8^

To achieve efficient directional water transport from the bulk solution to the evaporator and within the evaporator toward the evaporation interface, two strategies have proven effective: (1) introducing graded capillary channels to generate a capillary pressure gradient, and (2) anchoring polyelectrolytes in a gradient manner to establish an osmotic pressure difference. Graded capillarity can transport water at a rate of up to 0.072 g min^–1^;^13^ however, combined with gradient osmotic pressure, it achieves a 26% higher transport rate of 0.091 g min^–1^.^8^ When combined, the synergistic action of capillary and osmotic forces can be further enhanced through rational structural design, thereby maximizing water transport and evaporation efficiency.

## 4 Evaporative Surface Functionalization

Because evaporation occurs exclusively at the air–water interface, the evaporator surface plays a decisive role in determining efficiency. Under illumination, a photothermal evaporator absorbs light and converts it into heat, enabling water molecules to overcome intermolecular cohesion and vaporize (Figure 4a). This process not only depends on thermal energy generation but is also closely related to water association states. Prior studies have demonstrated that introducing functional groups capable of hydrophobic interaction, hydrogen-bonding, or electrostatic interaction with water can modulate its local water molecule orientation, weaken hydrogen-bonding, thereby lowering evaporation enthalpy and ultimately enhancing evaporation efficiency (Figure 4b).^6,8,9,18,43–48^ Rational structural and molecular design are thus indispensable—not only for optimizing energy harvesting and conversion, but also for tuning the evaporation enthalpy of water, both of which are critical for boosting overall evaporator performance.

**Figure 4:**
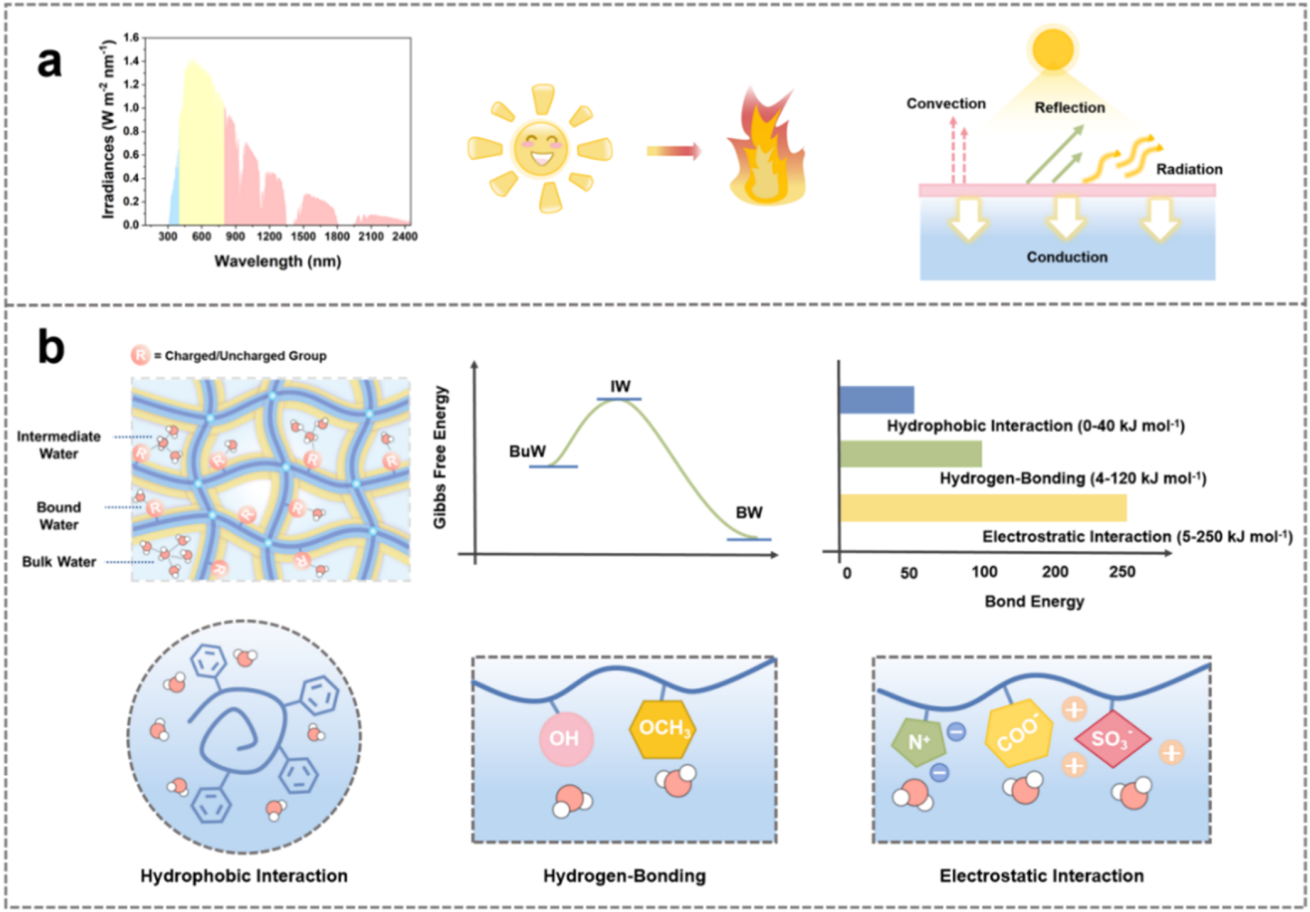
Energy harvesting and water-association-state regulation. (a) Broadband light absorption, efficient photothermal conversion, and thermal localization. (b) In the evaporative interface, three water association states coexist: intermediate water (IW), bound water (BW), and bulk water (BuW). IW has the highest free energy and is most prone to dissociation and evaporation, followed by BuW, whereas BW has the lowest free energy and is essentially nonevaporable. Surface interactions—including hydrophobic interaction (0–40 kJ mol^–1^), hydrogen-bonding (4–120 kJ mol^–1^), and electrostatic interaction (5–250 kJ mol^–1^)—orient interfacial water molecules and regulate these water association states, thereby reducing evaporation enthalpy and enhancing evaporation efficiency.

### 4.1 Energy Harvesting

Light absorption is the initial step of photothermal evaporation. Near-unity broadband absorptance across the solar spectrum (300–2500 nm, AM 1.5G) can be achieved through material hybridization and surface morphology engineering (i.e., waffle grids,^50^ vesicle-like pores,^29^ fish-scale patterns^200^). For example, Ququan Wang’s group^49^ hybridized Bi_2_Se_3_ with Cu_2‑*x*_S to leverage plasmon-enhanced semiconductor absorption, yielding an average absorptance of 94.3% across the solar band. Xuebin Wang’s group^50^ fabricated waffle-shaped carbonaceous evaporators via zinc-assisted pyrolysis, where multiple internal reflections or scattering suppressed reflectance, achieving 98.5% average absorptance.

Following light absorption, photothermal conversion efficiency directly governs evaporation performance. Importantly, not all incident optical energy is converted into heat; some is dissipated through nonthermal pathways (i.e., fluorescence, carrier separation), thereby reducing usable thermal energy. Liang Zuo’s group^27^ designed a flat-band λ-Ti_3_O_5_ semiconductor that suppresses carrier separation, achieving 92.4% photothermal conversion efficiency. The resulting 3D evaporator delivered an ultrahigh evaporation rate of 6.09 kg m^–2^ h^–1^.

Retaining generated heat at the evaporating interface is essential to avoid energy losses. In typical systems, heat loss is dominated by conduction (∼60%), followed by convection (∼30%) and radiation (∼10%). Conduction can be suppressed by placing low-thermal-conductivity porous insulators between the evaporator and bulk water, while convective losses can be mitigated by adopting 3D inverted-cone geometries that promote steam recirculation.

### 4.2 Energy Consumption

Interfacial evaporation is driven by heat input that activates water molecules for liquid–vapor transition. Beyond thermal energy supply, reducing evaporation enthalpy offers an effective route to accelerate evaporation.^17^ This requires regulating water association states—bound water (BW), bulk water (BuW), and intermediate water.^6,7^ BW forms tightly bound solvation layers and is hard to evaporate. BuW is stabilized by a four-hydrogen-bond network with high evaporation enthalpy (∼2400 J g^–1^).^47,51^ By contrast, IW has fewer hydrogen bonds and therefore exhibits lower evaporation enthalpy. Surface interaction—hydrophobic interaction, hydrogen-bonding, and electrostatic interaction—governs the distribution of water association states. Collectively, they enrich IW, reduce evaporation enthalpy, accelerate evaporation, and enhance salt rejection through ionic backflow, ion hydration competition, and co-ion repulsion ­(Figure 5, Supporting Table S1).^24,46,51–53^

**Figure 5:**
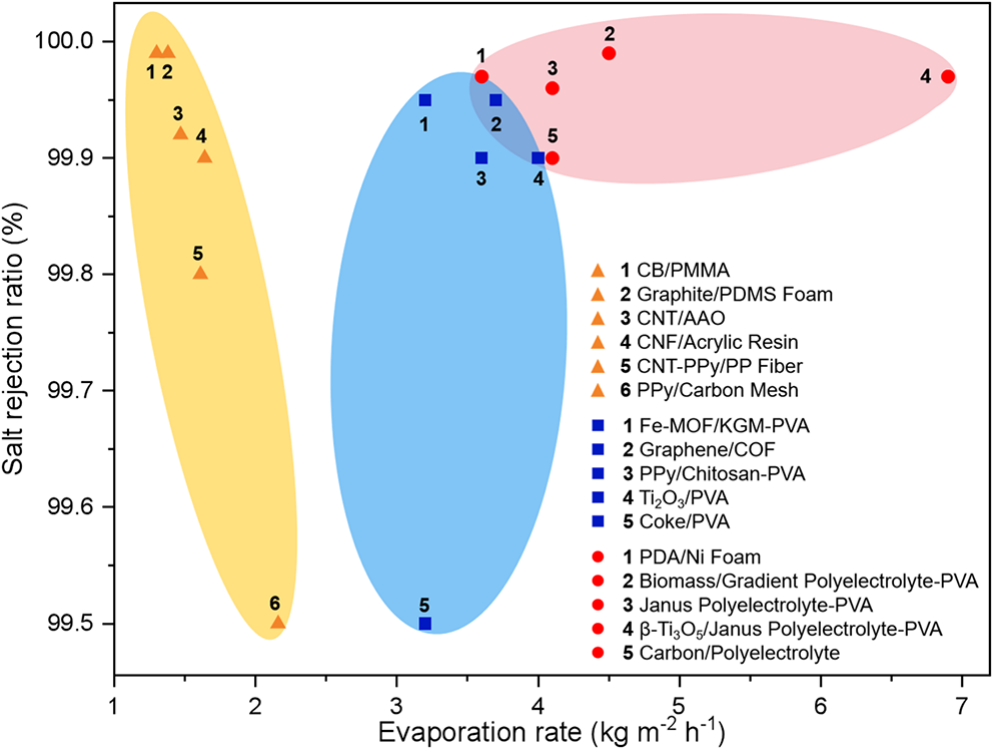
Comparison of evaporation rate and salt rejection ratio regulated by water association states through hydrophobic interaction (orange region), hydrogen-bonding (blue region), and electrostatic interaction (red region).^7–9,34,48,51,52,54–62^

Hydrophobic groups reduce water desorption enthalpy (0–40 kJ mol^–1^)^63^ and increase entropy, jointly accelerating water evaporation. Their incompatibility with ions further promotes ionic backflow, suppresses interfacial ion accumulation, and ensures salt resistance. Jia Zhu’s group^52^ constructed a carbon black/poly­(methyl methacrylate) (CB/PMMA) hydrophobic layer atop a polyacrylonitrile (PAN) nonwoven water-transport substrate via electrospinning and spray coating, achieving an evaporation rate of 1.3 kg m^–2^ h^–1^ under 1 kW m^–2^ illumination. The methyl groups of PMMA orient adjacent water molecules, weaken the local hydrogen-bonding, and enrich IW. Combined with the low water–methyl binding energy that facilitates desorption, this yields a favorable enthalpy–entropy synergy for evaporation. However, the weak interaction between the hydrophobic and hydrophilic layers compromises interfacial stability and structural robustness under seawater conditions.

To address this, Guihua Yu’s group^51^ covalently anchored microscale hydrophobic patches across the Ti_3_O_5_-based photothermal hydrogel via silane coupling, achieving an evaporation rate of 4.0 kg m^–2^ h^–1^ at 1 kW m^–2^ illumination. These patches elevate the interfacial free energy at the three-phase contact line, increasing local vapor pressure and promoting water evaporation, while hydrophobic–ion incompatibility suppresses salt transport to the interface, ensuring stable desalination. Nevertheless, microscale patches spanning the entire hydrogel restrict effective water transport pathways and limit the evaporation area. To overcome this, our group^47^ employed electric-field-assisted grafting to orderly arrange nanoscale core–shell polyelectrolyte micelles on the surface of PVA hydrogels, followed by good-solvent rinsing to form amphiphilic patch arrays (Figure 6). Remarkably, even without photothermal particles, the system achieved an evaporation rate of 3.2 kg m^–2^ h^–1^ under 1 kW m^–2^ illumination and 99.94% salt rejection in seawater desalination. Hydrophilic quanterized poly­(4-vinylpyridine) (P4VP) subpatches enabled osmotic pumping, while ∼40 nm polystyrene caps imparted nanoscale hydrophobicity. Compared with microscale patches, nanoscale patch arrays greatly increased the contact-line length. For instance, a microscale patch with 1 μm diameter provides a contact line length of 3.54 μm, whereas filling the same 1 μm^2^ area with closely packed 40 nm patches yields a total contact line length of ∼97 μm—about 27 times higher than that of the microscale case. This significant increase in contact-line length markedly enhances evaporation while preserving a large effective evaporative area and robust salt resistance.

**Figure 6:**
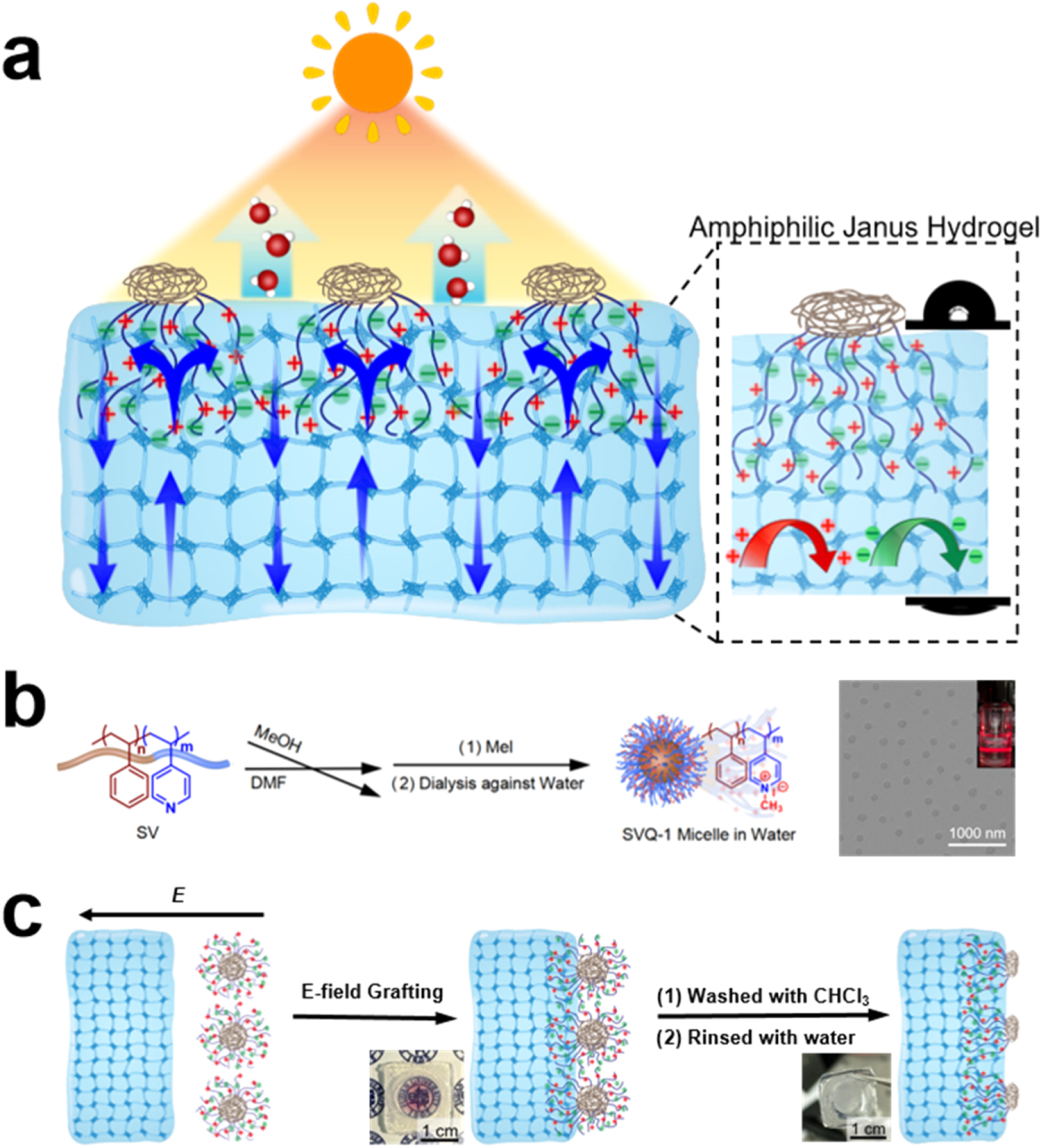
(a) Schematic illustration of an amphiphilic patch-surfaced hydrogel, facilitating water transport, evaporation, and salt rejection. (b) Fabrication of amphiphilic polyelectrolyte micelles and corresponding TEM image. (c) Grafting of amphiphilic micelles onto a hydrogel via an electric-field-assisted method. Reproduced with permission from ref 47. Copyright 2024, Royal Society of Chemistry.

Compared with hydrophobic interaction, hydrogen-bonding is a stronger, and directional noncovalent interaction (4 – 120 kJ mol^–1^)^64^ between a covalently bound hydrogen and an electronegative atom. Beyond water–water hydrogen-bonding, water can bond with polar groups such as hydroxyl and amine groups, reshaping water association state distribution and competing with ion hydration to enhance salt rejection.^65,66^ Guihua Yu’s group^7^ incorporated 1 wt % polypyrrole (PPy) as the photothermal component and systematically adjusted the PVA/chitosan ratio in hydrogels from 20:0 to 16:4, while maintaining a total polymer solid content of 8 wt %. This tuning shifted the IW/BuW ratios from 0.8 to 1.3. At the optimal PVA/chitosan ratio of 17:3, the evaporator achieved a peak evaporation rate of 3.6 kg m^–2^ h^–1^ with >99% salt rejection. The superior performance arises from cooperative hydrogen-bond engineering between hydroxyl groups in PVA and amine groups in chitosan, which act as donors/acceptors with distinct energetics. Their synergy perturbs the tetrahedral water network to enrich IW without excessive conversion to bound water, lowering the evaporation enthalpy from 2400 J g^–1^ of pure water to 900 J g^–1^ when measured in a dark-room experiment. Additionally, partially protonated amine groups of chitosan compete with ion hydration and induce a local Donnan exclusion, suppressing salt migration to the evaporating interface and enhancing stability.

Electrostatic interaction arises from Coulombic coupling between charged groups and water dipoles. By orienting water dipoles, charged groups enrich IW, lower the evaporation barrier, and simultaneously repel co-ions, thereby enhancing evaporation efficiency while suppressing interfacial salt accumulation for long-term stability. Compared with relatively weak and short-ranged hydrogen-bonding (4–120 kJ mol^–1^; 0.15–0.35 nm),^67,68^ electrostatic interaction is stronger and longer ranged (5–250 kJ mol^–1^; >10 nm),^69,70^ enabling regulation of a larger population of activated IW states. Guihua Yu’s group^62^ demonstrated that a poly­[2-(methacryloyloxy)­ethyl]­dimethyl-(3polysulfopropyl)­ammonium hydroxide (PDMAPS)-based photothermal hydrogel effectively disrupted the hydrogen-bond networks of water, yielding an IW/BuW ratio of 1.2 in 10 wt % brine and an evaporation rate of 4.14 kg m^–2^ h^–1^ under 1 kW m^–2^ illumination. Owing to electrostatic ion repulsion, the evaporator sustained this performance over 3 weeks of continuous operation in 10 wt % brine, exhibiting excellent salt-resistant stability. However, conventional strategies such as physical mixing or *in situ* polymerization generally fail to achieve high loading and controllable spatial distributions of polyelectrolytes in the evaporator, owing to like-charge repulsion among chains. Consequently, the interfacial area is underutilized, effective interaction sites with water are insufficient, and the nearly uniform osmotic field cannot sustain the gradients required for directional transport and ion exclusion. Therefore, realizing high evaporation rates with durable salt resistance requires advanced polyelectrolyte chemistry and rational structural design.

To overcome these limitations, our group introduced and, for the first time, realized biparental polyelectrolytes (i.e., quaternized polyvinylpyridine series, and quaternized poly­(2-(diethylamino)­ethyl methacrylate) series)^9,47,48^ that self-assemble into polyelectrolyte micelles with high chain grafting density, followed by electric-field grafting to construct ordered arrays on hydrogel surfaces (Figure 7a–c). This approach enables precise control of IW/BuW from 0.2 to 3.7.^47,48^ In photothermal-free systems, a record evaporation rate of 4.1 kg m^–2^ h^–1^ is achieved,^48^ while in photothermal systems, 100 h of continuous stable operation is realized with 6.92 kg m^–2^ h^–1^ and 99.97% salt rejection ratio.^9^ The nanosized polyelectrolyte micelles offer a chain grafting density up to 0.05 nm^–2^, creating high densities of charge sites and local osmotic platforms that markedly enhance osmotic pumping and suppress ion migration to the interface (Figure 7d). Simultaneously, quaternary ammonium (N^+^) centers generate tight hydration shells through electrostatic attraction, whereas the methyl groups (van der Waals radius ≈ 2 Å) locally repel water via hydrophobic interaction and steric effects (Figure 7e). Together, they form an interleaved electrostatic–hydrophobic potential field that efficiently perturbs interfacial hydrogen-bonding, reduces water–water cohesive energy, and increases the IW fraction. This molecular design and interfacial construction strategy overcomes the low-loading and uniform-osmotic-field limitations inherent to mixing or *in situ* systems. It achieves high polyelectrolyte loading, programmable spatial placement, and synergistic optimization of water association state and osmotic pumping, thereby reconciling high evaporation rate with ultrahigh salt rejection under long-term operation.

**Figure 7:**
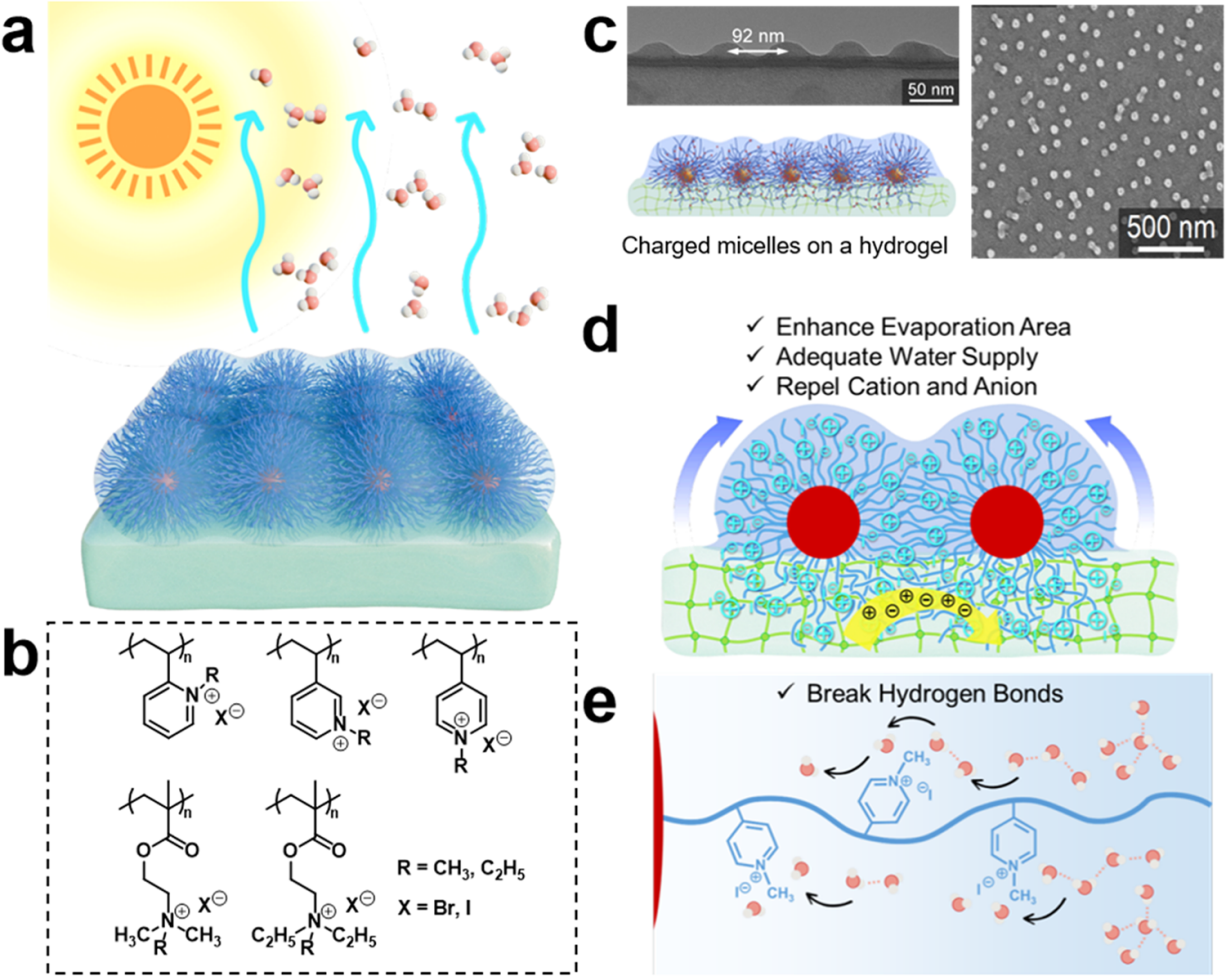
(a) Schematic illustration of a biparental polyelectrolyte array grafted on a hydrogel to enhance water evaporation. Reproduced with permission from ref 9. Copyright 2024, American Chemical Society. (b) Library of quaternized polyvinylpyridine and quaternized poly­(2-(diethylamino)­ethyl methacrylate) series. (c) TEM cross-section and SEM top-view images of biparental polyelectrolyte micelles, with a schematic illustration of micelles grafting on a hydrogel. Reproduced with permission from ref 48. Copyright 2025, Royal Society of Chemistry. (d) Mechanism of micelles facilitating evaporation by enlarging the interfacial area, supplying water, and repelling ions for salt rejection. Reproduced with permission from ref 9. Copyright 2024, American Chemical Society. (e) Role of methyl groups in disrupting hydrogen-bond networks via hydrophobic interaction and steric hindrance. Reproduced with permission from ref 9. Copyright 2024, American Chemical Society.

## 5 Conclusion and Outlook

The design of high-efficiency solar-driven interfacial evaporators fundamentally relies on three elements: floating capability, effective water transport, and precisely engineered evaporative surfaces. Floating capability ensures reliable operation, but must be coupled with thermal insulation and efficient water transport to sustain high evaporation performance. Efficient water transport maintains a steady water supply to the evaporative surface, ensuring stable vapor generation. Meanwhile, surface engineering regulates light absorption, photothermal conversion, and thermal confinement for optimized heat management, while simultaneously tailoring interfacial properties to modulate water association states, lower the evaporation barrier, and enhance both evaporation rate and salt resistance.

To further expand the functional potential of solar evaporators, these systems can be integrated with complementary energy-harvesting technologies. The heat generated during evaporation can be utilized to drive thermoelectric modules, converting temperature gradients into electrical energy. Likewise, the salinity gradients produced during evaporation can be exploited for salinity-gradient power generation. In addition, incorporating photocatalysts into the evaporative surface enables photocatalytic reactions, such as hydrogen generation or pollutant degradation, thereby broadening the range of applications for solar-driven evaporators.

Despite these advances, a precise mechanistic understanding of interfacial evaporation remains limited. Conventional thermal analysis (i.e., differential scanning calorimetry (DSC)) and spectroscopic methods (i.e., Raman spectroscopy) often suffer from bulk-signal interference, obscuring the molecular-level processes at the interface. The development of high-sensitivity, low-noise interfacial characterization techniques will therefore be critical for resolving evaporation mechanisms, bridging theory with experiment, and guiding rational design principles for next-generation devices.

From a practical perspective, despite the remarkable advantages of evaporators, the absence of unified design standards and the reliance on complex materials and architectures have resulted in costly, challenging fabrication that limits scalability. Industrial deployment demands simplified designs and scalable, low-cost fabrication, where the balance between practicality and performance defines both the challenge and the opportunity for bringing solar evaporation into commercial reality.

## Supplementary Material



## Notes

Summary of evaporation rates and salt rejection ratios (PDF)

## Abbreviations

AAO: anodic aluminum oxide
AM 1.5G: air mass global
BuW: bulk water
BW: bound water
CB: carbon black
CNF: carbon nanofiber
CNT: carbon nanotube
COF: covalent organic framework
DSC: differential scanning calorimetry
IW: intermediate water
KGM: konjac glucomannan
MOF: metal organic framework
N^+^: quaternary ammonium
PAANa: sodium poly­(acrylic acid)
PAN: polyacrylonitrile
PDA: polydopamine
PDMAPS: poly­[2-(methacryloyloxy)­ethyl]­dimethyl-(3polysulfopropyl)­ammonium hydroxide
PDMS: polydimethylsiloxane
PMMA: poly­(methyl methacrylate)
PP: polypropylene
PPy: polypyrrole
PS: polystyrene
PVA: poly­(vinyl alcohol)
SEM: scanning electron microscope
TEM: transmission electron microscope
