# Supplementary material for: From Structural Design to Molecular Mechanisms: The Evolution of Solar Evaporators
Source: Chem Bio Eng. 2025 Dec 9;3(2):148–58. doi: 10.1021/cbe.5c00110 (PMC12951244; doi:10.1021/cbe.5c00110)
Supplement: Supplementary file 1 [file be5c00110_si_001.pdf]

Supporting Information

From Structural Design to Molecular Mechanisms: The Evolution of Solar Evaporators

Dong Wu, Jie Zhu, Qichen Zhang, Xiayun Huang*, Zhihong Nie*

Address: The State Key Laboratory of Molecular Engineering of Polymers and Department of Macromolecular Science, Fudan University, Shanghai 200438, People's Republic of China

Email: huangxiayun@fudan.edu.cn (Xiayun Huang); znie@fudan.edu.cn (Zhihong Nie)

Contents

Supplementary Table S1

References

Supplementary Table

Table S1: Summary of evaporation rates and salt rejection ratios

Evaporators' name[a]	Preparation method, Composition, and structure of the evaporator	Main interactions	Salt rejection ratio (%)	Evaporation rate (kg m^{-2} h^{-1})	Ref.
CNT-PPy/PP Fiber	Dip coating and spray coating. PP non-woven, CNTs, PPy. 2D monolithic membrane	Hydrophobic interaction	99.80	1.61	[1]
Graphite/PDMS Foam	Dip coating. Graphite, melamine foam, PDMS. Janus 3D monolithic aerogel.	Hydrophobic interaction	99.99	1.38	[2]
CNF/Acrylic Resin	Doping and photopolymerization. CNF, Acrylic resin. Arch-shaped monolith.	Hydrophobic interaction	99.90	1.64	[3]
PPy/Carbon Mesh	Template-assisted chemical deposition. PPy, Carbon mesh. Platform-assisted 2D membrane	Hydrophobic interaction	99.50	2.16	[4]
CNT/AAO	Template-assisted chemical vapor deposition. CNT, AAO. Platform-assisted 2D membrane	Hydrophobic interaction	99.92	1.47	[5]
CB/PMMA	Electrospinning and spray coating. CB, PMMA, PAN. 2D monolithic membrane	Hydrophobic interaction	99.99	1.3	[6]

Coke/PVA	Doping and freeze-melting.<br>Coke, PVA.<br>3D monolithic hydrogel.	Hydrogen-bonding	99.50	3.2	[7]
Fe-MOF/KGM/PVA	Magnetic-field-assisted gelation.<br>Fe-MOF, KGM, PVA<br>3D monolithic hydrogel.	Hydrogen-bonding	99.95	3.2	[8]
Graphene/COF	Hydrothermal method.<br>Graphene, COF-SO_3H.<br>3D monolithic hydrogel.	Hydrogen-bonding	99.95	3.7	[9]
PPy/Chitosan-PVA	*In situ* cogelation.<br>PPy, Chitosan, PVA<br>Janus 3D monolithic hydrogel.	Hydrogen-bonding	99.90	3.6	[10]
Ti_2O_3/PVA	Gelation and silane modification.<br>Ti_2O_3, PVA, silane reagent.<br>Janus 3D monolithic hydrogel.	Hydrogen-bonding	99.90	4	[11]
Carbon/Polyelectrolyte	Polymerization and gelation.<br>Activated carbon, polyeletrolyte.<br>3D monolithic hydrogel.	Electrostatic interaction	99.90	4.1	[12]
PDA/Ni foam	*In situ* polymerization and dip coating.<br>PDA, Ni foam, sporopollenin.<br>3D monolithic aerogel.	Electrostatic interaction	99.97	3.6	[13]
β-Ti_3O_5/Janus Polyelectrolyte-PVA	Gelation and eletric-field-assisted grafting.<br>β-Ti_3O_5, PVA, polyeletrolyte.<br>Janus 3D monolithic hydrogel.	Electrostatic interaction	99.97	6.9	[14]
Janus Polyelectrolyte-PVA	Gelation and eletric-field-assisted grafting.<br>PVA, polyeletrolyte.<br>Janus 3D monolithic hydrogel.	Electrostatic interaction	99.62	4.1	[15]

Biomass/Gradient Polyelectrolyte-PVA	Gelation and eletric-field-assisted grafting. PVA, polyeletrolyte. Gradient 3D monolithic hydrogel	Electrostatic interaction	99.99	4.5	[16]

[a] A/B: A denotes the photothermal materials and B denotes the functional materials to regulate

References

(1) Shuangjie Sun; Yameng Wang; Binbin Sun; Feifei Zhang; Qing Xu; Hao Yang Mi; Heng Li; Xiaoming Tao; Zhanhu Guo; Chuntai Liu; Changyu Shen. Versatile Janus composite nonwoven solar absorbers with salt resistance for efficient wastewater purification and desalination, *ACS Appl. Mater. Interfaces*, **2021**, *13*, 24945-24956.
(2) Jinxing Chen; Jessica Lujia Yin; Bo Li; Zuyang Ye; Dilong Liu; Deng Ding; Fang Qian; Nosang Vincent Myung; Qiao Zhang; Yadong Yin. Janus evaporators with self-recovering hydrophobicity for salt-rejecting interfacial solar desalination, *ACS Nano*, **2020**, *14*, 17419-17427.
(3) Miaomiao Zou; Yu Zhang; Zheren Cai; Chuxin Li; Zhiyuan Sun; Cunlong Yu; Zhichao Dong; Lei Wu; Yanlin Song. 3D printing a biomimetic bridge-arch solar evaporator for eliminating salt accumulation with desalination and agricultural applications, *Adv. Mater.*, **2021**, *33*, 2102443.
(4) Zhen Yu; Ruonan Gu; Yue Tian; Pengfei Xie; Beichen Jin; Shaoan Cheng. Enhanced interfacial solar evaporation through formation of micro-meniscuses and microdroplets to reduce evaporation enthalpy, *Adv. Funct. Mater.*, **2022**, *32*, 2108586.
(5) Qiancheng Xia; Yifan Pan; Bin Liu; Xin Zhang; Enze Li; Tao Shen; Shuang Li; Ning Xu; Jie Ding; Chao Wang; Chad D. Vecitis; Guandao Gao. Solar-driven abnormal evaporation of nanoconfined water, *Sci. Adv.*, **2024**, *10*, eadj3760.
(6) Weichao Xu; Xiaozhen Hu; Shendong Zhuang; Yuxi Wang; Xiuqiang Li; Lin Zhou; Shining Zhu; Jia Zhu. Flexible and salt resistant Janus absorbers by electrospinning for stable and efficient solar desalination, *Adv. Energy Mater.*, **2018**, *8*, 1702884.
(7) Hongqi Zou; Xiangtong Meng; Xin Zhao; Jieshan Qiu. Hofmeister effect-enhanced hydration chemistry of hydrogel for high-efficiency solar-driven interfacial desalination, *Adv. Mater.*, **2023**, *35*, 2207262.
(8) Youhong Guo; Hengyi Lu; Fei Zhao; Xingyi Zhou; Wen Shi; Guihua Yu. Biomass-derived hybrid hydrogel evaporators for cost-effective solar water purification, *Adv. Mater.*, **2020**, *32*, 1907061.
(9) Changxia Li; Sijia Cao; Jana Lutzki; Jin Yang; Thomas Konegger; Freddy Kleitz; Arne Thomas. A covalent organic framework/graphene dual-region hydrogel for enhanced solar-driven water generation, *J. Am. Chem. Soc.*, **2022**, *144*, 3083-3090.
(10) Xingyi Zhou; Fei Zhao; Youhong Guo; Brian Rosenberger; Guihua Yu. Architecting highly hydratable polymer networks to tune the water state for solar water purification, *Sci. Adv.*, **2019**, *5*, eaaw5484.
(11) Youhong Guo; Xiao Zhao; Fei Zhao; Zihao Jiao; Xingyi Zhou; Guihua Yu. Tailoring surface wetting states for ultrafast solar-driven water evaporation, *Energy Environ. Sci.*, **2020**, *13*, 2087-2095.
(12) Chuxin Lei; Weixin Guan; Youhong Guo; Wen Shi; Yuyang Wang; Keith P. Johnston; Guihua Yu. Polyzwitterionic hydrogels for highly efficient high salinity solar desalination, *Angew. Chem. Int. Ed.*, **2022**, *61*, e202208487.
(13) Yi Wang; Weinan Zhao; Yebin Lee; Yuning Li; Zuankai Wang; Kam Chiu Tam. Thermo-adaptive interfacial solar evaporation enhanced by dynamic water gating, *Nat. Commun.*, **2024**, *15*, 6157.
(14) Jie Zhu; Xiaoya Zhao; Naibing Wu; Dong Wu; Xiayun Huang; Zhihong Nie; Daoyong Chen. Constructing a thin layer of the concentrated polyelectrolyte solution on the hydrogel surface that can considerably improve the solar evaporation efficiency, *Macromolecules*, **2024**, *57*, 9811-9822.

(15) Jie Zhu; Dong Wu; Xiayun Huang; Daoyong Chen; Zhihong Nie. Hydrogen-bond disruption in molecularly engineered Janus evaporators for enhanced solar desalination, *Soft Matter*, **2025**, *21*, 2114-2123.
(16) Jie Zhu; Shaoen Qiu; Mingyu Duan; Qihao Xie; Oushuo Jiang; Xinran Zhao; Dong Wu; Yaxi Liu; Guang Chen; Xiayun Huang; Zhihong Nie. Polyelectrolyte gradient hydrogels for efficient solar evaporation, *Adv. Funct. Mater.*, **2025**, e12350.
